# Pakistan’s Healthcare System: A Review of Major Challenges and the First Comprehensive Universal Health Coverage Initiative

**DOI:** 10.7759/cureus.44641

**Published:** 2023-09-04

**Authors:** Salman J Khan, Muhammad Asif, Sadia Aslam, Wahab J Khan, Syed A Hamza

**Affiliations:** 1 Hematology and Oncology, Mayo Clinic, Jacksonville, USA; 2 Public Health, University of Massachusetts, Amherst, USA; 3 Internal Medicine, Avera McKennan Hospital and University Health Center, Sioux Falls, USA; 4 Internal Medicine, University of South Dakota Sanford School of Medicine, Sioux Falls, USA; 5 Cardiology, Punjab Institute of Cardiology, Lahore, PAK

**Keywords:** healthcare delivery system, health care disparity, health system challenges, health policy making, universal health coverage (uhc), pakistan's healthcare system

## Abstract

Each country's healthcare system has a different structure and functioning designed to meet the needs of its people utilizing the available resources. Due to ever-growing population needs and constantly emerging public health problems, it is vital for any healthcare system to be ready to adapt, recognize its limitations, and improve its flaws by learning from other healthcare models across the globe. In this article, we analyzed the significant challenges faced by Pakistan's healthcare system (PHS) and the first comprehensive initiative taken for universal health coverage in Pakistan. Inequitable distribution of resources, inadequate healthcare spending, non-adherence to preventative healthcare and brain drain are the major problems in the PHS. On the other hand, the recently introduced universal health coverage initiative, the Sehat Sahulat Program (SSP), can be considered one of the biggest achievements of the country’s healthcare system.

## Editorial

Introduction

No healthcare system can be labeled as perfect because of the growing needs of people, constantly emerging new public health challenges, and the diversity of population demographics around the globe. Every system needs continuous pruning to fulfill the needs of its people through analysis of its shortcomings and strengths. Pakistan's healthcare system (PHS) is not an exception to this principle. PHS comprises private and public sectors, catering to a huge population of more than 220 million [1]. There are many challenges faced by PHS including inadequate funding, infrastructural limitations, brain drain of health professionals, limited focus on preventive healthcare (PHC), and inequitable resource allocation. Among these issues, Pakistan's first comprehensive universal health coverage (UHC) initiative, Sehat Sahulat Program (SSP), can be considered the most outstanding achievement of the PHS.

Challenges

PHS faces many challenges that hinder its ability to provide adequate and efficient healthcare services to its citizens. One of the significant challenges is insufficient funding. Pakistan spends around 38 US Dollars (USD) per capita on healthcare, which is much lower than other developing countries [2]. As compared to Pakistan, India, the Philippines, and Ghana spend 57, 165, and 85 USD per capita on healthcare, respectively [2]. Pakistan spent 1.2% of its gross domestic product (GDP) on the public health sector in 2020-2021 as compared to 1.1 in 2019-2020, which is not a significant increase when viewed in terms of GDP percentage [3]. The lack of sufficient investment in the PHS has led to another challenge which is a shortage of health infrastructure, medicines, medical equipment, and qualified healthcare professionals. Although there is an increase in human resources from 2014 to 2021, this growth is not enough to cater to the needs of the population growing at 2% per annum (Table 1) [3]. Around 32,879 physicians graduate every year in Pakistan and 40% of them go abroad for better opportunities citing low income, long hours of job, and inequality as the main reasons [4]. According to a study conducted at two different medical colleges, 33% of medical students plan to leave the country to practice healthcare abroad. This brain drain puts undue pressure on the PHS resulting in inadequate provision of health facilities to people.

**Table 1:** Human Resources in the Pakistan's Healthcare System Open access source: Pakistan Economic Survey 2021-22 [3]

Health Manpower	2017	2018	2019	2020	2021
Doctors	208,007	220,829	233,261	245,987	266,430
Dentists	20,463	22,595	24,930	27,360	30,501
Nurses	103,777	108,474	112,123	116,659	121,245
Midwives	38,060	40,272	41,810	43,129	44,693
Lady Health Workers	18,400	19,910	20,565	21,361	22,408

Limited focus on PHC is another significant issue PHS faces. PHC includes measures to prevent diseases and promote health, such as immunizations, screenings, and health education. Pakistan's government has taken several steps over the years to promote PHC which include the Lady Health Workers (LHW) programme, the expanded programme on immunization (EPI), the Polio Eradication Initiative (PEI) Programme, the Malaria Control Programme (MCP), Tuberculosis (TB), Control Programme, and establishment of basic health units (BHUs) and rural health units and rural health units (RHUs). In 2021, there were 1,276 hospitals, 5,558 BHUs, 736 RHCs, 5,802 Dispensaries, 780 Maternity and Child Health Centers, and 416 TB centers in Pakistan [3]. However, all these initiatives have not been able to drastically improve the health indicators of Pakistan, which are much worse than its peers (Table 2). These initiatives are not enough for a population of more than 220 million [1]. There is still a scarcity of resources in the PHC realm and the people do not have access to these services because of less developed PHC centers or even the absence of these centers nearby. Even with access to these facilities, the population does not get involved in preventive health because of a lack of awareness and education regarding its importance to their own health. Many people in Pakistan lack basic health literacy, which means they do not have the knowledge and skills to access and use healthcare services effectively.

**Table 2:** Health Indicators of Pakistan Open access source: Economic Survey of Pakistan [3]

Health Indicators of Pakistan		
	2019	2020
Maternal Mortality Ratio (Per 100,000 Births)	189	186
Neonatal Mortality Rate (Per 1,000 Live Births)	41.2	40.4
Under-5 Mortality Rate (Per 1,000)	67.3	65.2
Incidence of Tuberculosis (Per 100,000 People)	263	259
Life Expectancy at Birth (Years)	67.3	67.4
Births Attended By Skilled Health Staff (% of Total)	68.0	69.3
Source: WDI, UNICEF, Trading Economics & Our World in data		

The inequitable distribution of healthcare resources is a serious threat to the PHS. The healthcare resources, including hospitals, clinics, and healthcare professionals, are concentrated in the urban areas, leaving rural areas with inadequate healthcare facilities. It leads to a significant disparity in healthcare access and outcomes between urban and rural populations [5]. The Community Health Index (CHI) reflects the unequal distribution of healthcare resources. CHI measures the disparities between different regions based on health and well-being. Pakistan scored an inequality ratio of 16.59 CHI, which means that the upper-tier districts are 16.59 times healthier than the lower-tier districts. The disparity ratio differs by approximately 10 points between the urban and rural areas (7.78 and 17.54, respectively) showing a huge disparity in resources. This data reveals the inequitable distribution of resources in the healthcare domain in Pakistan [5]. Consequently, the rural healthcare system lacks basic medical equipment, diagnostic facilities, and medications, leading to a lack of proper patient diagnosis and treatment. All these shortages increase the burden on the infrastructure in cities and, in turn, lead to inadequate provision of health facilities, physician shortages, and dissatisfaction among patients.

Sehat Sahulat Program: UHC Initiative

UHC is a concept coined by the WHO that aims to ensure essential health services to everyone without any financial hardship. UHC is a part of the Sustainable Development Goals (SDGs) adopted by the United Nations (UN) in 2015. Pakistan is a signatory of the SDGs. The goal of UHC is expressed in the UN 2030 agenda as part of the SDGs in Goal 3, which focuses on health (target 3.8). UHC is the primary step toward providing health as a fundamental right of citizens [2]. The biggest achievement of the PHS is the UHC initiative in the form of the SSP.

SSP is a public sector-funded health insurance initiative of the federal and provincial governments working to provide financial health protection to all citizens against extraordinary healthcare expenditure. SSP is a landmark healthcare initiative that is considered an important step toward UHC. SSP was implemented first by the Khyber Pakhtunkhwa (KPK) provincial government in 2015 to provide free health insurance coverage to the poor and vulnerable populations only. Then, the federal government of Pakistan in cooperation with the provincial governments rolled out the SSP in other provinces in 2019. The program is funded by the government of Pakistan and is managed by the Ministry of National Health Services, Regulations, and Coordination. The program has two main components: (i) free health insurance coverage for eligible households and (ii) a network of participating hospitals and clinics where eligible households can access healthcare services. The SSP initially provided social insurance only to families living below the poverty line but is now gradually moving toward every citizen. As of 2022, the SSP has been implemented in 36 districts of Punjab, 35 districts of Khyber Pakhtunkhwa, 10 districts of Azad Jammu and Kashmir (AJK), 10 districts of Gilgit Baltistan (GB), Islamabad Capital Territory (ICT) and Hardaker district of Sindh, reaching approximately 44.6 million households. The Public Sector Development Program (PSDP) is responsible for contributing premiums from ICT, AJK, GB, Federally Administered Tribal Areas (FATA), and Thar Parker districts. However, Punjab and KP fund 100% premium contributions from various sources [3].

Under the SSP, households receive health insurance cards, which can be used to access healthcare services up to one million rupees per year at participating hospitals and clinics. The program covers a wide range of inpatient services, including cardiac procedures, cancer management, burn management, dialysis, complications of diabetes mellitus, trauma management, neurosurgical procedures, abdominal surgeries, fracture management, and other medical and surgical interventions [3]. The program has a tiered benefit structure with higher benefits for households with more vulnerable members, such as women, children, and older people. The SSP has a vast network of more than 1030 paneled hospitals across Pakistan. Beneficiaries from any district can get treatment from any of these paneled hospitals. The program has also positively impacted the financial protection of marginalized communities. Transgender people and persons with disabilities registered with the National Database Regulatory Authority (NADRA) were also enrolled in this program. They have given access to UHC, a giant leap in the inclusion of the ignored community [3]. In Pakistan, out-of-pocket (OOP) expenditures on health are more than 60% of the total health expenditure [3]. The SSP has shared this cost at every level of healthcare. Moreover, it serves 154 million people in Pakistan, which is the first-ever health insurance initiative in the history of Pakistan [3]. Till March 8, 2022, over 3.2 million hospital visits have been recorded under the SSP's health cards [1]. The shared health expenditure has also facilitated people's access to medical services which they used to avoid in the past due to high healthcare costs, thus promoting health and wellness.

There are a few limitations to this program. Many families have complained about the incompatibility between the cost of treatment in private-sector hospitals and the limits set by the program. Patients are expected to pay the difference. In some instances, patients were turned away without any medical services due to the inability to pay [1]. Another issue is the interrupted continuity of the SSP due to recent political and economic instability in Pakistan. It is still functional in some parts of the country while being suspended in others.

Conclusion

As Pakistan is a developing country, its healthcare system must make many improvements to meet the needs of its population. The challenges faced by Pakistan's healthcare system include insufficient funding, inadequate healthcare workforce and infrastructure, less focus on preventive health, and inequitable distribution of resources. These challenges need comprehensive policy formulation focused on increases in healthcare funding and allocation of equity-based resources. The most significant achievement of PHS is the initiative toward UHC through the SSP. This initiative has decreased the burden of healthcare expenses and increased access to healthcare services for people, including marginalized communities.
